# Copper(II) benzyloxychalcone analogues as new potential metallodrugs against SARS-CoV-2 replication

**DOI:** 10.1099/jgv.0.002245

**Published:** 2026-05-05

**Authors:** Igor Andrade Santos, Victoria Riquena Grosche, Laiane dos Santos Oliveira, Pedro Henrique de Souza Guarda, Gustavo Clauss Rodrigues, Rayany Cristina de Souza, Deborah Cristina Teixeira Alves, Andres Merits, Robinson Sabino-Silva, Dalan Bailey, Camilla Abbehausen, Mark Harris, Ana Carolina Gomes Jardim

**Affiliations:** 1 Institute of Biomedical Sciences, Federal University of Uberlândia, Uberlândia, Brazil; 2 School of Molecular and Cellular Biology, Faculty of Biological Sciences, University of Leeds, Leeds, LS2 9JT, UK; 3 Viral Glycoproteins Group, The Pirbright Institute, Pirbright, Woking, UK; 4 Institute of Biosciences, Humanities and Exact Sciences, São Paulo State University, São Paulo, Brazil; 5 Institute of Chemistry, University of Campinas, Campinas, Brazil; 6 Innovation Centre in Salivary Diagnostics and Nanobiotechnology, Laboratory of Nanobiotechnology – “Luiz Ricardo Goulart”, Department of Physiology, Institute of Biomedical Sciences, Federal University of Uberlandia, Uberlandia, Brazil; 7 Institute of Bioengineering, University of Tartu, Tartu, Estonia

**Keywords:** antivirals, Cu(II) complexes, coronavirus disease 2019 (COVID-19), chalcones, severe acute respiratory syndrome coronavirus 2 (SARS-CoV-2)

## Abstract

Chalcones, a naturally occurring class of molecules found in various plants, serve as both precursors and final products in the biosynthesis of flavonoids. Renowned for their diverse therapeutic actions, chalcones demonstrate anti-inflammatory, antitumoral, antimalarial and antiviral activities. The structure of chalcones allows chemical manipulation, making them attractive for metal coordination, such as with copper, an essential metal for living organisms. Here, we characterize the activity of CuL_2_phen and CuL_1_phen against severe acute respiratory syndrome coronavirus 2 (SARS-CoV-2), in which L1 and L2 are two forms of the chalcones 3-(4-(benzyloxy)phenyl)-1-(4-fluoro-2-hydroxyphenyl)prop-2-en-1-one and 3-(4-(benzyloxy)phenyl)-1-(2-hydroxyphenyl)prop-2-en-1-one, respectively, and phen is phenanthroline. CuL_1_phen and CuL_2_phen anti-SARS-CoV-2 activity were studied in the viral replication cycle employing both the SARS-CoV-2-NeonGreen infectious clone and wild-type isolates. The SI of CuL_1_phen and CuL_2_phen was found to be 1.7 and 5.5, respectively, demonstrating that CuL_2_phen is a more promising compound. CuL_2_phen impaired SARS-CoV-2 entry, predicted by molecular docking calculations to disrupt the glycoprotein S and angiotensin-converting enzyme 2 (ACE2) binding, emphasized by the low EC_50_ in pseudotyped virus entry assay. Further, CuL_2_phen was identified as SARS-CoV-2 post-entry inhibitor, probably due to its strong interaction with SARS-CoV-2 double stranded RNA. Altogether, the data suggest that CuL_2_phen acts by impairing SARS-CoV-2 entry by disrupting the viral envelope as well as interrupting RNA replication through specifically intercalating into the dsRNA. The obtained results give us mechanistic insights into the activity of this promising Cu(II) metallodrug candidate in SARS-CoV-2 infection.

**Impact Statement**

This study expands the literature on chalcone-based metallodrugs by demonstrating the therapeutic potential of copper(II) complexes in combating severe acute respiratory syndrome coronavirus 2 (SARS-CoV-2). While chalcones are widely recognized for their diverse biological activities, this work uniquely integrates their chemical versatility with copper coordination to develop compounds targeting a pressing global health challenge. The findings bridge a gap in understanding how such compounds interact with viral components at various stages of the replication cycle, providing a mechanistic perspective on their antiviral action. The breadth of interest lies in the dual mechanism of CuL_2_phen, which not only disrupts viral envelope and entry processes but also interferes with RNA replication via dsRNA interactions. This demonstrates its potential in different areas, such as virology, drug design and medicinal chemistry. Furthermore, the inhibition profile highlights the relevance of copper-based chalcones as promising candidates in antiviral drug development and represents a significant incremental advance, delivering mechanistic insights that could inform the rational design of next-generation metallodrugs with enhanced efficacy against SARS-CoV-2 and other RNA viruses. This work lays a foundation for exploring the broader applicability of chalcone-based copper complexes in antiviral therapeutics.

## Data Summary

The authors confirm that all supporting data, code and protocols have been provided within the article or through supplementary data files. The full sequence of the SARS-CoV-2 wild-type used in this manuscript is available in the National Center for Biotechnology Information (NCBI) Sequence Read Archive (project ID PRJNA1368746, sequence ID SAMN53368779).

## Introduction

Severe acute respiratory syndrome coronavirus 2 (SARS-CoV-2) and its disease, coronavirus disease 2019 (COVID-19), continue to burden health systems and impact social and economic aspects in many countries around the world [1,4]. Up to date, over 771 million confirmed cases and 6.9 million COVID-19-related fatalities have been reported globally, of which 37.7 million cases and 704,000 deaths have been reported in Brazil [5]. After vaccination, the number of severe cases and deaths decreased [6]; however, the emergence of new variants continues to facilitate virus spread [1,2]. Of concern, SARS-CoV-2-infected individuals can develop post-COVID-19 syndrome (long COVID-19), which is characterized by long-lasting symptoms that can persist for months after infection [7,9].

Currently, three antiviral drugs have been approved by regulatory agencies for treating COVID-19 patients: remdesivir, molnupiravir and Paxlovid^®^ [10,13]. However, these drugs are exclusively prescribed for severe COVID-19 cases, with the clinical management of mild to moderate symptoms using analgesics and anti-inflammatory drugs [14,15]. In this scenario, the limited number of direct-acting antivirals and the restriction of treatment to severe cases may potentially render untreated patients more susceptible to the development of post-COVID-19 syndrome [7,9].

Chalcones are naturally occurring molecules found in various plants, serving as both precursors and final products in the biosynthesis of flavonoids [16]. These molecules can be readily synthesized on a large scale and are amenable to straightforward chemical modification, resulting in a wide array of synthetic derivatives [17,19]. Their basic structure comprises two aromatic rings linked by an *α*,*β*-unsaturated ketone moiety, forming 1,3-diaryl-2-propen-1-one in a planar skeleton [18]. This structure can be modified by adding groups to the aromatic rings, consequently changing their properties. Chalcones have been described for their anti-inflammatory, antitumoral, antimalarial and antiviral activities through different mechanisms of action [17,20,25]. Given their previous reported activity and potential chemical manipulation, chalcones are compelling candidates for metal coordination.

Metal coordination involves the chemical binding of organic or inorganic molecules to a metal ion, such as copper (Cu), platinum (Pt), palladium (Pd), ruthenium (Ru) and silver (Ag), among others, through a Lewis acid-base reaction known as a coordination bond [26,27]. Interestingly, when combined with specific classes of ligands, these new metal-based compounds can address the multidimensionality of several diseases, including cancer [28,29], Alzheimer’s disease [30] and viral [31,32] and bacterial infections [33,34]. This approach is marked by the The Food and Drug Administration (FDA) approval of cisplatin [cis-diaminodichloridoplatin(II)] for the treatment of several types of cancer, about 50 years ago [29,35]. Since then, a whole research field has been dedicated to designing and studying a plethora of metallic complexes based on Pt, Au, Ru, Ag, Cu and other metals [32].

Among the metal ions of interest, Cu(II) holds significance as an essential dietary metal for living organisms, capable of boosting the host immune system and serving as a cofactor for important enzymes [36,37]. Additionally, it exhibits antiviral, antifungal and antibacterial activity as a salt [36,38,40]. Cu(II) can primarily act by damaging the viral envelope [41,42], generating reactive oxygen species or interacting with the DNA or RNA of the virus [41,45]. Coordinating Cu(II) to biocompatible ligands can improve selectivity, biodistribution and enhance the activity of the drug, thereby decreasing dose-limiting toxicity [46,47]. With this in mind, our group recently designed and evaluated the effect of the chalcones 3-(4-(benzyloxy)phenyl)-1-(4-fluoro-2-hydroxyphenyl)prop-2-en-1-one as L_1_ and 3-(4-(benzyloxy)phenyl)-1-(2-hydroxyphenyl)prop-2-en-1-one as L_2_, along with the Cu(II) complexes Cu(L_1_)_2_, Cu(L_2_)_2_ CuL_1_phen and CuL_2_phen against the SARS-CoV-2 [48]. Remarkably, CuL_1_phen and CuL_2_phen inhibited around 85% SARS-CoV-2 replication at 2 µM, while their precursors L_1_ and L_2_ showed no activity against this virus [48].

**Fig. 1.**
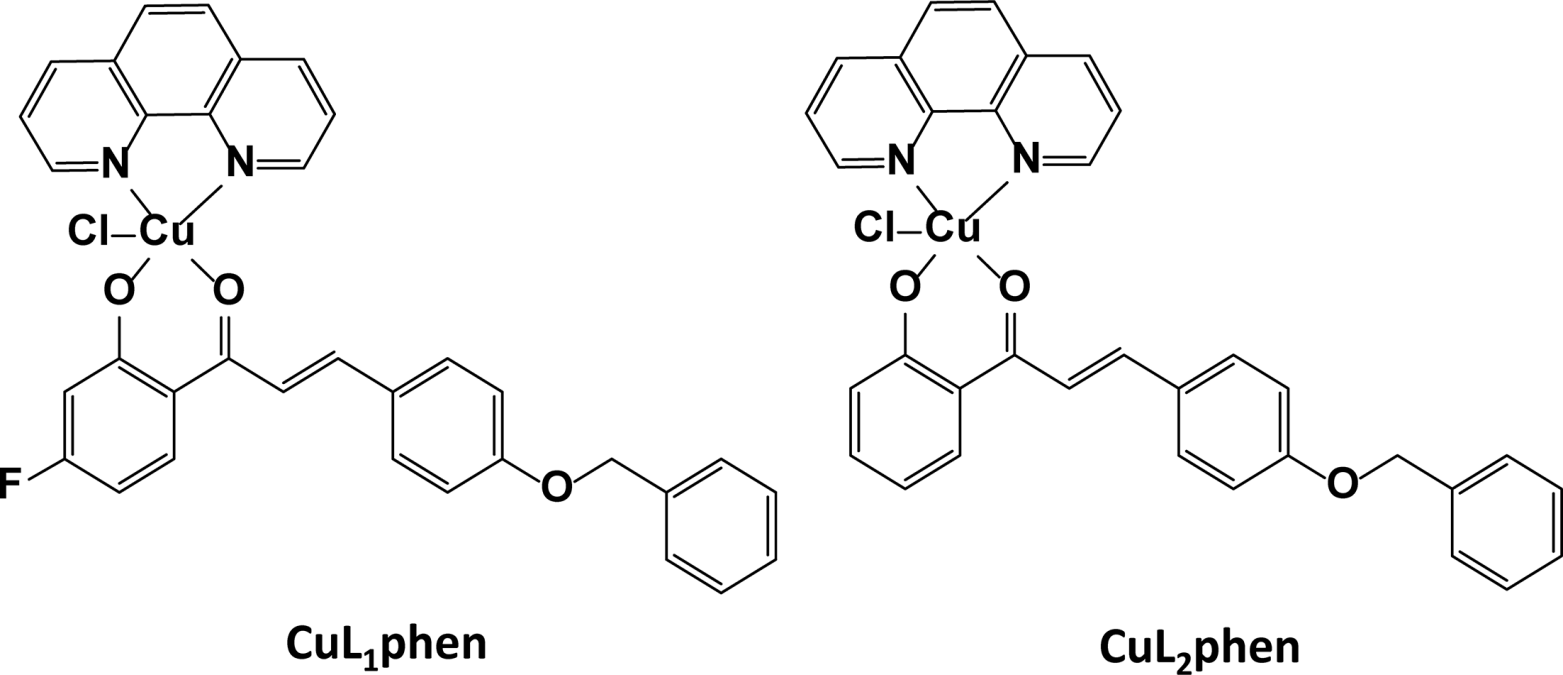
Chemical structure of CuL_2_phen and CuL_1_phen [48].

Here, we further characterized the activity of these new Cu(II) complexes, CuL_1_phen and CuL_2_phen (Fig. 1), against SARS-CoV-2, evaluated their action on different stages of the virus replicative cycle and suggested one potential mode of action for these complexes.

## Methods

### Cell culture and compounds

Human adenocarcinoma alveolar basal epithelial cells expressing angiotensin-converting enzyme 2 (ACE2) and TMPRSS2 receptors [A549-AT, National Institute for Biological Standards and Control (NIBSC), UK, #101004], African green monkey kidney cells expressing ACE2 and TMPRSS2 (Vero-E6-ACE2-TMPRSS2, NIBSC, UK, #101003), baby hamster kidney cells (BHK-21, ATCC #CCL-10) [48] and human embryonic kidney 293T, HEK293T cells (ATCC^®^, catalogue number: CRL-3216^TM^) were cultivated using Dulbecco’s Modified Eagle’s Medium (DMEM) (Sigma-Aldrich) supplemented with 100 U ml^−1^ penicillin (Gibco Life Technologies), 100 mg ml^−1^ streptomycin (Gibco Life Technologies), 1% (v/v) non-essential amino acids (Gibco Life Technologies) and 10% (v/v) FBS (Hyclone) at 37 °C in a humidified 5% CO_2_ incubator. The cells were cultivated in the presence of Geneticin (G418) and Hygromycin B at 1 mg ml^−1^ and 200 µg ml^−1^, respectively [49]. HEK293T cells expressing ACE2 receptor were cultivated as described above with the addition of puromycin at 4 µg ml^−1^.

The metal complexes CuL_2_phen and CuL_1_phen (Fig. 1) were synthesized as previously described [48], where L_1_ is [3-(4-(benzyloxy)phenyl)-1-(4-fluoro-2-hydroxyphenyl)prop-2-en-1-one and L_2_ is 3-(4-(benzyloxy)phenyl)-1-(2-hydroxyphenyl)prop-2-en-1-one, both deprotonated, and phen is phenanthroline. All the compounds were dissolved in DMSO to a stock concentration of 50 mM prior to the assays, stored at −20 °C for a maximum of 2 days and diluted in cell culture media immediately prior to the assays to a final concentration of 0.1% (w/v).

### Viruses

The parental Wuhan-like (hCov-119/England/02/2020, GISAID access number: EPI_ISL_407073), B.1.617.2 - Delta (MS066352H, GISAID access number EPI_ISL_1731019) and BA.2 – Omicron (hCoV/England/FCI-179/2022, [50]) viruses were isolated and characterized by the Francis Crick Institute and kindly provided by Professor Stephen Griffin at the University of Leeds. The viruses were amplified by infecting Vero E6-ACE2-TMPRSS2 cells in a 175 cm² flask until 80% of cytopathic effect 3 days post-infection (d.p.i.). Supernatant was collected, and the infectious titre was determined using the TCID_50_ method and calculated by the Spearman and Kärber algorithm as described [51]. The titre was further converted to p.f.u. ml^−1^ using the formula p.f.u. ml^−1^=0.69 × TCID_50_ [52,53]. After amplifying the parental Wuhan-like SARS-CoV-2 used in this study, the supernatant was collected, the vRNA extracted using QIAmp Viral RNA Kit and submitted to sequencing as previously described [54], and the full sequence is now publicly available in the National Center for Biotechnology Information (NCBI) Sequence Read Archive (project ID PRJNA1368746, sequence ID SAMN53368779). The genome sequence was compared to SARS-CoV-2 Wuhan-like (NC_045512.2) as described in [55]. The identified mutations, frequency and position in SARS-CoV-2 genome are available at Table S1.

### Rescue of infectious virus

The rescue of SARS-CoV-2 reporter virus based on the NCBI sequence NC_045512.2 was performed as previously described [48,56]. Briefly, a total of 1 µg of the infectious clone pCCI-4K-SARS-CoV-2-NeonGreen under the CMV promoter [56,57] was transfected into BHK-21 (3×10^5^ cells per well in a 6-well plate) using Lipofectamine 2000 (1:). After 3 days, the supernatant was collected and transferred to A549-AT cells in a T75 flask until complete cell lysis (3 d.p.i), and the supernatant was harvested as P0 stock. The infectious titre was determined using the TCID_50_ method, calculated by the Spearman and Kärber algorithm as described [51], and the titre was further converted to pfu/mL using the formula p.f.u. ml^−1^=0.69 × TCID_50_ [52,53].

### Pseudotyped virus generation, titration and purification

Pseudotyped viruses (PVs) carrying the S protein of either B.1, B.1.617.2 or BA.2 SARS-CoV-2 were generated as previously described with modifications [58,59]. HEK293T cells were seeded in 6-well plates at a density of 8×10^5^ cells per well. After 24 h, a mix of 600 ng of p.891 HIV Gag-Pol, 600 ng pCSFLW luciferase and 500 ng of either B.1, B.1.617.2 or BA.2 SARS-CoV-2 spike in pcDNA3.1 vector was prepared in 100 µl of Optimen followed by the addition of PEI at 1 mg ml^−1^ for 20 min. The pcDNA3.1 plasmid and the Vesicular Stomatitis Virus glycoprotein (VSV‑G) were used as the empty vector and pseudotyping control, respectively. The solution was added to the culture media dropwise. Cell culture media was changed 24 h after transfection, and supernatant was collected at 48 h and 72 h post-transfection, centrifuged at 2,500 r.p.m. for 10 min, aliquoted and stored at −80 °C.

PVs were titrated using a tenfold serial dilution in DMEM, followed by the addition to HEK293T-ACE2 cells at 2×10^4^ cells per well in a 96-well plate. After 72 h, luciferase levels were measured by adding Bright-Glo reagent (Promega) and reading in GloMAx luminometer (Promega). Quantification was based on relative luminescence units per millilitre (RLU ml^−1^).

To confirm pseudotyping, an aliquot of supernatant corresponding to each transfection and the empty vector was purified by ultracentrifugation (Optima XPN-100 Ultracentrifuge, Beckman Coulter) for 55,000 r.p.m. for 1 h at 4 °C in a 20% sucrose cushion using polypropylene tubes (#326819, Beckman Coulter). After centrifugation, the supernatant was discarded, and pelleted PVs were resuspended in 0.22 µm filtered PBS. Sixty microlitres of the PVs resuspension was diluted in Laemmli 1× (Bio-Rad) containing beta-mercaptoethanol (Thermo-Fisher) and reduced for 10 min at 95 °C. Samples were resolved on 12.5% acrylamide gels in SDS-PAGE and transferred onto a nitrocellulose membrane using a semidry transfer. Blots were probed for S protein using the mouse anti-spike 1A9 (1:1,000, #GTX632604, GeneTex) and the goat anti-HIV p24 (1:1,000, #AB19961, Abcam) in PBS 5% milk powder (w/v) for 1 h at room temperature, washed with PBS 0.1% Tween, followed by secondary anti-mouse HRP (#7076, Cell Signalling) and rabbit anti-goat HRP (#81-1620, Invitrogen) both at 1:2,000 for 1 h at room temperature. Membranes were exposed using the Clarity Western ECL substrate (Bio-Rad) following the manufacturer’s instructions.

### Dose–response assay

To evaluate the cytotoxicity and activity profiles of CuL_2_phen and CuL_1_phen on cell viability and viral replication, A549-AT cells were seeded at a density of 1×10^4^ cells per well into 96-well plates 24 h prior to the infection and incubated at 37 °C and 5% CO_2_. Then, cells were treated with each complex in a twofold serial dilution, with concentrations ranging between 0.58 and 300 µM, in the presence or absence of SARS-CoV-2-NeonGreen at an MOI of 0.1, as previously described [56,57]. Cell viability was measured by 3-(4,5-dimethylthiazol-2-yl)-2,5-diphenyl tetrazolium bromide (MTT) (Sigma-Aldrich) assay, as previously described [48]. After treatment, media containing the compound were removed from each well and MTT at 1 mg ml^−1^ solution was added, incubated for 30 min and replaced with 100 µl of DMSO to solubilize the formazan crystals. The absorbance was measured at 570 nm on FLUOstar OPTIMA microplate reader (BMGLabTech). For the antiviral assays, the plates were placed on the IncuCyte^®^ S3 Live-Cell Analysis System (Sartorius) after 24 h, and green fluorescence was observed at 10× objective. The photos were analysed employing the basic analyser from the IncuCyte S3 system and the total integrated intensity of the fluorescence (GCU × µm^2^ per well) was collected.

To evaluate the effect of CuL_2_phen on A549-AT and HEK293T-ACE2 cytotoxicity and SARS-CoV-2 entry, both cell lines were seeded at 2×10^4^ cells per well in 96-well plates 24 h prior to treatment. CuL_2_phen was diluted in a twofold serial dilution, with concentrations ranging between 0.78 and 200 µM, in the presence of each PV at 10^6^ RLUs per well and incubated for 1 h at 37 °C, then added to the cells and incubated for 72 h at 37 °C and 5% CO_2_. The luciferase levels were measured using Bright-Glo (Promega) and cell viability by MTT 72 h after treatment, as described above.

Cell viability and viral replication/entry were calculated according to the equation (*T*/*C*)×100%, where *T* and *C* represent the optical density of the treated well and control groups, respectively. Cells treated with DMSO 0.1% were used as the mock-treated control. The effective concentration of 50% (EC_50_) and cytotoxic concentration of 50% (CC_50_) were calculated employing a non-linear regression. The selective index (SI) was calculated by the equation, SI=(CC_50_/EC_50_)

### Time of drug addition assays

A549-AT cells were seeded in 96-well plates at a concentration of 1×10^4^ cells per well, 24 h prior to the infection and incubated at 37 °C and 5% CO_2_. Treatment was carried out with CuL_2_phen at 2 µM, and the extent of viral replication was quantified based on the intensity of green fluorescence when observed through a 10× objective lens. The acquired images were subsequently subjected to analysis using the basic analyser integrated within the IncuCyte S3 system, which calculated the total integrated fluorescence intensity, measured in GCU × µm^2^ per well.

In the pretreatment assay, cells were treated for 1 h with the complexes before being infected with the SARS-CoV-2-NeonGreen at a multiplicity of infection (MOI) of 0.1. Subsequently, the cells were thoroughly washed with PBS, and the unbound virus was removed by incubation with fresh media for 24 h (37 °C and 5% CO_2_).

In the entry inhibition assay, cells were infected (MOI of 1) and simultaneously treated with the complexes for 1 h. After that, cells were washed with PBS and then incubated with fresh media for 24 h (37 °C and 5% CO_2_). To evaluate virucidal activity, a similar approach was used, except that the complex and virus mixture (MOI of 5) was incubated for 1 h before being added to the cells.

To investigate the impact of the compound on the attachment step of the viral infection, the same setting as in the entry inhibition assay was employed, wherein the cells were incubated with the virus (MOI of 1) and the compound at 4 °C. Another variation of this protocol was performed by incubating the cells and the compounds with the virus at 4 °C for 1 h and then at 37 °C for a 30-min incubation to assess the effect of the compound on post-attachment steps of infection.

In the post-entry assay, cells were infected with the SARS-CoV-2-NeonGreen virus at an MOI of 0.1 for 1 h, followed by thorough washing with PBS. Subsequently, they were incubated in a medium containing the complex for 24 h. Additionally, a variation of this assay involved infecting the cells with the virus at an MOI of 0.1 for 6 h and then treating the infected cells with the complex for the remaining duration to evaluate its effects after the establishment of viral replication, here described as RNA replication.

The MOI in each experimental setting was increased to ensure synchronization of infection, a high number of cells infected, and to avoid multiple rounds of re-infection, enabling an accurate assessment of early events, such as attachment, entry and uncoating [60,61] or decreased for later stages, to avoid saturation effects and allow for clearer observation of downstream replication events.

### Virion formation and release assay

Quantification of viral RNA in both supernatant and cell lysates was performed as previously described [49,62]. Briefly, cells were infected with SARS-CoV-2 Wuhan-like (hCov-119/England/02/2020) at an MOI of 0.1 and treated with CuL_2_phen at 2 µM for 24 h, with cells treated with DMSO 0.1% being the mock-treated control. After incubation, supernatants and cell lysates from treated and mock-treated infected cultures were collected separately. Total RNA was extracted using TRIzol^®^ reagent (Invitrogen, Waltham, MA, USA), converted to cDNA with the LunaScript^®^ RT SuperMix Kit (New England Biolabs, Hitchin, UK) and quantified by real-time quantitative PCR (qPCR) using the GoTaq^®^ qPCR Master Mix (Promega, Southampton, UK), as described in [49,62]. Viral RNA copy numbers were calculated by comparison to a standard curve and reported as log10 values. The standard curve was generated from a tenfold serial dilution of cDNA derived from SARS-CoV-2 supernatant at 10⁶ p.f.u. ml^−1^ (1.45×TCID_₅₀_ ml^−1^). For cell lysate samples, viral RNA quantification was performed using the same method, with results normalized to GAPDH mRNA levels obtained using specific primers. All real-time qPCR reactions were conducted in duplicate, including both negative controls (nuclease-free water) and positive controls (SARS-CoV-2-mCherry cDNA).

### Replication of SARS-CoV-2 subgenomic replicon in BHK-21 cells

To assess the effect of CuL_2_phen on RNA replication and translation, the SARS-CoV-2 subgenomic replicon plasmid pCCL-4K-SARS-CoV-2-Repl-PL-NLuc based on the SARS-CoV-2 Wuhan strain genome was used, and a replication assay was performed as previously described [49]. Briefly, BHK-21 cells were seeded in 48-well plates at 5×10^4^ cells per well, and after 24 h, cells were transfected with 600 ng of the plasmid followed by treatment with CuL_2_phen at 2 µM. Cells treated with DMSO 0.1% were used as the mock-treated control. After 72-h incubation, cells were lysed with passive lysis buffer, and luminescence levels were quantified with the Nano-Glo^®^ Luciferase Assay System (Promega, Southampton, UK). Cell viability assays were carried out simultaneously. Viral replication and cell viability were calculated according to the equation (*T*/*C*)×100%, in which *T* and *C* represent the optical density of the treated well and control groups, respectively.

### Broad-spectrum activity against SARS-CoV-2 variants

To evaluate the impact of CuL_2_phen on the Delta and Omicron variants of SARS-CoV-2, an infectious clone originating from the SARS-CoV-2 Wuhan strain, which includes a MCherry reporter gene, was genetically modified to replace the spike gene sequence with those derived from the B.1.617.2 (Delta) and BA.2 (Omicron) variants. The rescue of these genetically altered viruses followed the protocols previously described [49,56]. For antiviral screening purposes, CuL_2_phen at 2 µM was added to the culture media containing the Wuhan-B.1.617.2-Spike or Wuhan-BA.2-Spike viruses at an MOI of 0.1, and this combination was then transferred to the cells for a duration of 24 h. The quantification of viral replication was conducted by measuring the total integrated fluorescence intensity, expressed as RCU×µm^2^ per well, employing the IncuCyte S3 microscope.

For the antiviral assays employing the wild-type viruses B.1.617.2 - Delta (MS066352H) and BA.2 – Omicron (hCoV/England/FCI-179/2022), A549-AT cells were cultured at a density of 8×10^5^ cells in 12-well plates and infected with each virus at an MOI of 0.1 in the presence or absence of CuL_2_phen at 2 µM for 24 h. The supernatant was collected, and the infectious titre was determined as described above.

### CuL_2_phen interaction assay with SARS-CoV-2 dsRNA

To investigate the potential interaction of CuL_2_phen with dsRNA, a gel retardation assay was conducted following previously described protocols [63,65], with modifications. The SARS-CoV-2 ORF1a was amplified with primers flanked by a T7 promoter site by PCR (forward: *TAATACGACTCACTATAGGG*GACCGAAAGGTAAGATGGAG; reverse:*TAATACGACTCACTATAGGG*AAATCGCCCGTCTGCCATGAAG; T7 promoter sites underlined). The reaction product of 499 bp was purified by Monarch^®^ PCR and DNA Cleanup Kit (New England BioLabs^®^) and used for *in vitro* transcription by the HiScribe™ T7 High Yield RNA Synthesis Kit (New England BioLabs^®^). The dsRNA molecule was obtained by complementary annealing in annealing buffer (potassium acetate 2M, Hepes-KOH 1M and magnesium acetate 1M) at 95 °C for 1 min. The SARS-CoV-2 dsRNA was quantified using NanoDrop (ThermoScientific) and incubated at 65 nM with CuL_2_phen at 2 µM for 45 min, and then the samples were analysed in 1% agarose 1X TAE gel stained with SYBR™ Safe DNA Gel Stain (Invitrogen). The absence or decrease of band intensity in the agarose gel suggests the compound interaction activity since it competes with SYBR. *cis*-[PtCl_2_(dmso)_2_] (Pt, 10 µM) was used as a positive control of interaction [64]. The band quantification was performed using ImageJ.JS version 1.53 j.

### Attenuated total reflection coupled to Fourier transform infrared analysis

The infrared spectra were obtained using a Benchtop attenuated total reflection (ATR)-Fourier transform infrared (FTIR) spectrometer (Agilent Cary 630 FTIR, Agilent Technologies, Santa Clara, CA, USA). The platform is equipped with a diamond ATR that performs an internal reflection to capture the infrared signature with a wavenumber range from 4,000 to 650 cm^−1^ in Microlab PC software. To this assay, solutions containing SARS-CoV-2 Wuhan-like (5×10^3^ p.f.u. or 7.2×10^3^ TCID_50_ ml^−1^), CuL_2_phen and SARS-CoV-2 Wuhan-like (5×10^3^ p.f.u. 7.2×10^3^ TCID_50_ ml^−1^) and CuL_2_phen were incubated for 1 h at 37 °C and 5% CO_2_. Then, all samples (3 µl) were inserted directly into the diamond and dehydrated to remove water for 12 min using constant airflow and humidity until each sample formed a thin dry layer on the ATR crystal. Each spectrum was then recorded with a resolution of 2 cm^−1^, 64 scans and 3,596 points per spectrum (0.931 cm^−1^ of spectral resolution). Before each analysis, a meticulous cleaning process of the crystal was conducted with 70% alcohol to prevent any inter-sample contamination. The biofingerprint of the second derivative spectrum was analysed from 3,050 to 2,800 cm^−1^ attached in 1,800–900 cm^−1^ in the Origin Pro^®^ 2021 9.8.0.200 (OriginLab, Northampton, MA, USA) software and adjusted using the Savitzky–Golay algorithm with polynomial order 2 and 21 points of the window (66 [62]).

### Molecular docking

The structures of [Cu(L_2_)(phen)Cl] and [Cu(L_2_)(phen)]^+^ were optimized using DFT with the ORCA software version 5.0.1 [67]. The PBE0 functional [68], def2-TZVP basis function [69], def2/J auxiliary basis, RIJCOSX approximation [70], CPCM for implicit DMSO solvation and a convergence criterion of 1.0×10^−8^ a.u. were employed [71]. Geometry optimizations were verified by frequency calculations at the same level of theory. The [Cu(L_2_)(phen)Cl] structure was compared with its single crystal as previously described [48].

To understand the interaction of these complexes with the receptor-binding domain (RBD) subunit of the SARS-CoV-2 spike protein, their optimized structures were docked against the isolated RBD (extracted from PDB: 7E86) [72] and RBD–ACE2 complex (PDB: 8T25) [73]. The BD-508 Fab was manually removed from PDB 7E86 using Discovery Studio Visualizer v21.1.0.20298 software before docking.

GOLD software was used for docking calculations with the genetic algorithm (GA) and ChemPL scoring function [74]. Ten GA runs were performed for each ligand, and the docking binding site was centred on the BS1 hotspot [75] (L455, F456, F486, N487, Y489 and Q493) with exact coordinates *x*: −26.1250, *y*: −29.7620, *z*: −0.8180 for the isolated RBD and *x*: 274.2551, *y*: 252.8858, *z*: 230.1930 for the RBD–ACE2 complex. The search window was adjusted to 2.857×Rg [76], resulting in ~20 Å for both.

The best poses from each calculation were chosen for further analysis. Protein–protein affinity calculations of the RBD and ACE2, with and without the docked complexes, were conducted via the Molecular Mechanics/Generalized Born Surface Area (MM/GBSA) function [77,78] implemented in the HawkDock web Server [79], using the ff02 force field, implicit solvent model and GBOBC1 model.

### Statistical analysis

Antiviral assays were performed in a minimum of 2 individual experiments (*n*=2) in quadruplicate to confirm the reproducibility of the results. All datasets were submitted to normality and log-normality tests to identify if the data were parametric or non-parametric. Differences between means of readings were compared using Student’s t-test. *P*-values of<0.05 (indicated by asterisks) were considered statistically significant. For the establishment of EC_50_ and CC_50_ values, the data were transformed into Log(*X*), where *X* is the concentration, and submitted to a non-linear regression with four parameters in variable slope. All analyses were performed using GraphPad Prism 9.

## Results

### CuL_2_phen possesses a higher selective index against SARS-CoV-2 replication

**Fig. 2.**
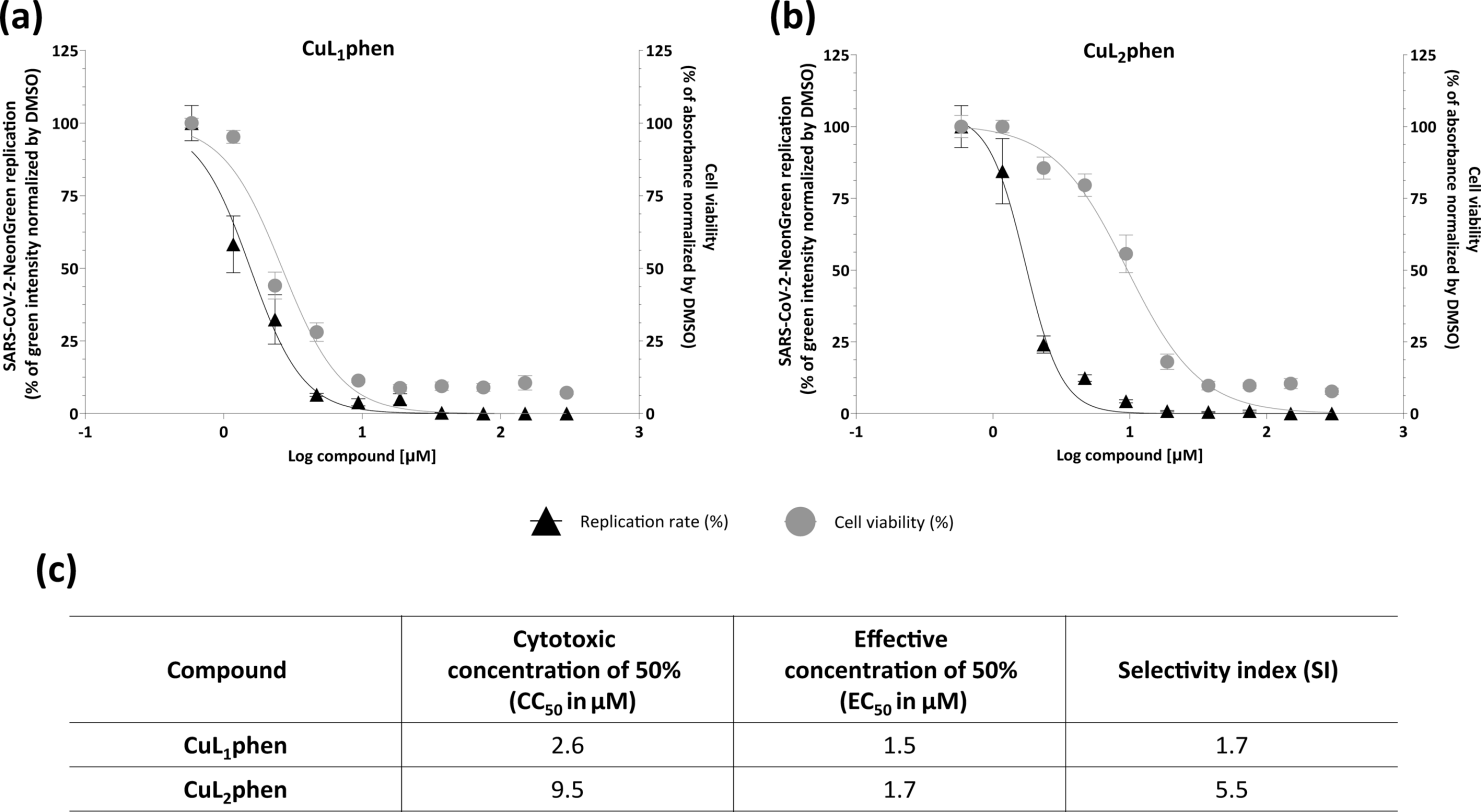
Dose–response curve of CuL_2_phen and CuL_1_phen on A549-AT cells and SARS-CoV-2-NeonGreen replication. A549-AT cells were infected with SARS-CoV-2-Neongreen at an MOI of 0.1 and treated with CuL_2_phen (**a**) or CuL_1_phen (**b**) at concentrations ranging from 0.58 to 300 µM, in twofold serial dilutions. After 24 h, the total integrated intensity of the fluorescence (GCU×µm^2^ per well) was analysed using the IncuCyte S3 microscope. Cell viability was assessed by treating A549-AT cells with the compounds in the same concentrations. After treatment, the media with compound was removed and replaced with media with MTT at 1 mg ml^−1^ and incubated for 30 min. After incubation, the media were removed, and the crystals were solubilized with DMSO. Surviving cells were measured by absorbance (570 nm). Mean values of two independent experiments, each measured in quadruplicate, including the standard deviation are shown. The EC_50_, CC_50_ and SI are shown in (**c**). All images were generated using GraphPad Prism 9 and Adobe Illustrator 2025.

Since our group recently described preliminary data on the anti-SARS-CoV-2 activity of CuL_2_phen and CuL_1_phen [48], here we aimed to further investigate their cytotoxicity and antiviral profiles, determining the CC_50_ and EC_50_, respectively. A549-AT cells treated with CuL_2_phen and CuL_1_phen at concentrations ranging from 0.58 µM to 300 µM in a two-serial dilution were infected with SARS-CoV-2-Neongreen at an MOI of 0.1. After 24 h, viral replication and cytotoxicity were assessed by the fluorescence intensity and MTT assay, respectively. This analysis showed that CuL_2_phen had a CC_50_ of 9.5±1.16 µM and EC_50_ of 1.7±0.16 µM (*P*<0.0001, *F*_3, 147_=147.1), resulting in a SI of 5.5 (Fig. 2a), whereas CuL_1_phen had a CC_50_ and EC_50_ of 2.6±0.32 µM and 1.5±0.21 µM (*P*<0.0001, *F*_2,156_=19.9), respectively, with a SI of 1.7 (Fig. 2b, c). Considering the higher SI of CuL_2_phen, further assays were only conducted with CuL_2_phen at 2 µM.

### CuL_2_phen restricts SARS-CoV-2 entry steps in A549-AT cells

**Fig. 3.**
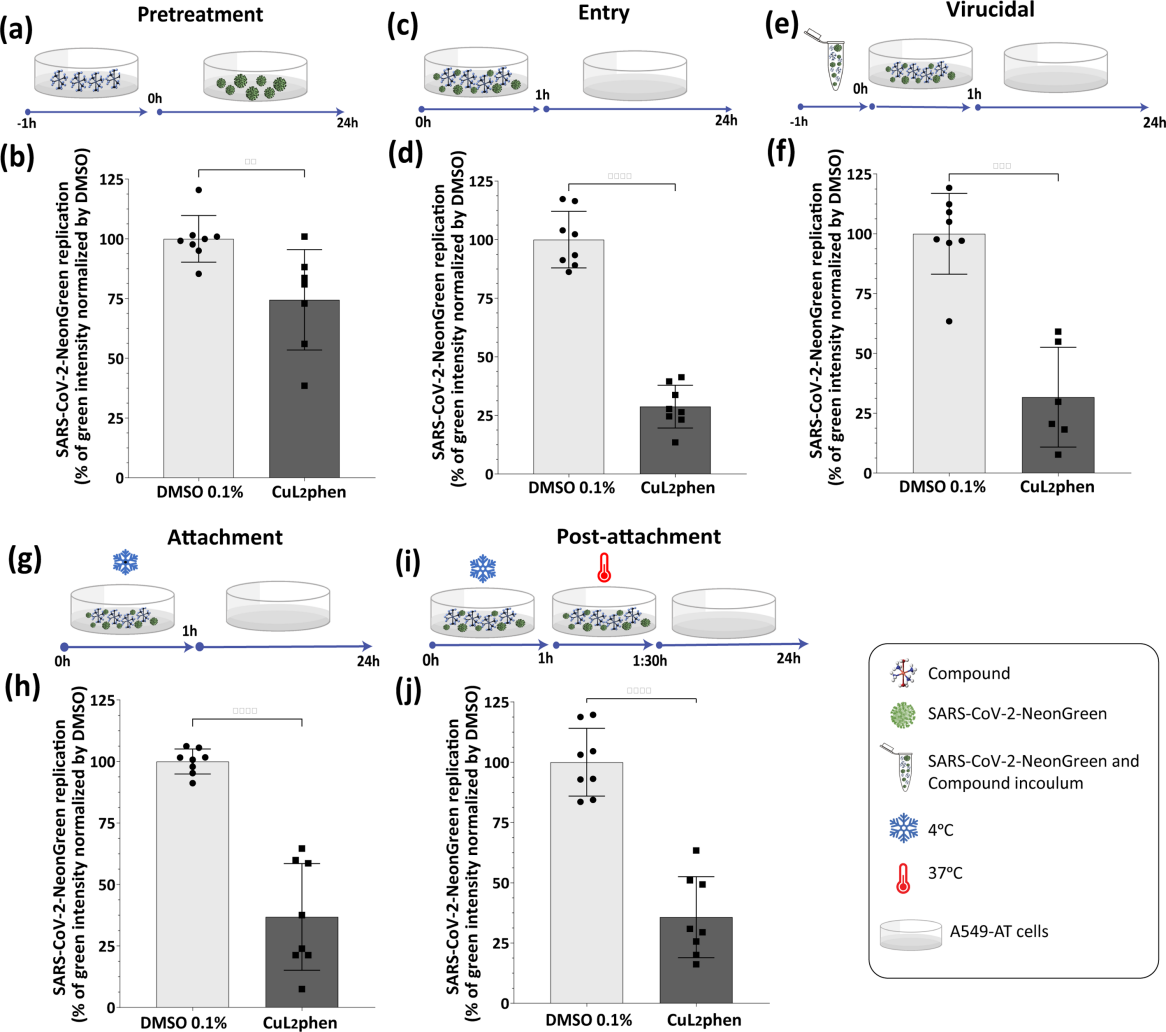
CuL_2_phen effect on SARS-CoV-2 infection. (**a and b**) A549-AT cells were treated with CuL_2_phen at 2 µM for 1 h. Then, cells were washed and infected with SARS-CoV-2-NeonGreen at an MOI of 0.1. (**c and d**) A549-AT cells were infected with SARS-CoV-2-NeonGreen (MOI 1) and simultaneously treated with CuL_2_phen at 2 µM for 1 h. Cells were washed and replaced with fresh media. (**e and f**) SARS-CoV-2-NeonGreen (MOI 5) and each compound were incubated for 1 h at 37 °C and then for one extra hour with the cells. Then, the virus and compound were removed, the cells were washed with PBS and fresh media were added. (**g and h**) A549-AT cells were infected with the virus and simultaneously treated with each compound for 1 h at 4 °C. The cells were washed to remove the virus and compound and replaced with fresh media. (**i and j**) A549-AT cells were infected with the virus and simultaneously treated with the compounds for 1 h at 4 °C. Then, cells were incubated for an additional 30 min with the compound and virus at 37 °C, washed with PBS and replaced with fresh media. For all assays, the total integrated intensity of the fluorescence (GCU×µm^2^ per well) was analysed using the IncuCyte S3 microscope. Schematic representation of each time-based assay as indicated by A549-AT cells (green arrows), compounds (chemical structure), SARS-CoV-2-NeonGreen (green virus), SARS-CoV-2-NeonGreen and compound inoculum (microtube), incubation at 4 °C (ice crystal) and incubation at 37 °C (thermometer). Mean values±sd of a minimum of three independent experiments, each measured in quadruplicate. Statistical differences were calculated by Student's t-test. (***) *P*<0.001, (****) *P* < 0.0001. All images were generated using GraphPad Prism 9 and Adobe Illustrator 2025.

To investigate the effect of CuL_2_phen on the replicative cycle of SARS-CoV-2, a time-of-drug-addition assay was conducted. Initially, to assess its protective effects against infection, A549-AT cells were treated with CuL_2_phen for 1 h, followed by SARS-CoV-2-NeonGreen infection at an MOI of 0.1 for 1 h then replaced with fresh media (Fig. 3a). As a result, CuL_2_phen demonstrated a 25.6% reduction in replication (Fig. 3b, *P*<0.0086), indicating this might not be the primary mechanism of its activity.

Due to the low impact of CuL_2_phen in protecting cells against SARS-CoV-2 infection, its effect was further investigated at different entry steps of SARS-CoV-2. Firstly, SARS-CoV-2-NeonGreen at an MOI of 0.1 and CuL_2_phen were concurrently added to the cells for 1 h at 37 °C (Fig. 3c), and showed 71.3% inhibition of viral entry (Fig. 3d, *P*<0.0001). The variation of this assay in which treatment was combined with an additional 1 h pre-incubation of the inoculum (Fig. 3e), resulted in a 56.6% reduction in viral infection (Fig. 3f, *P*=0.0002).

The strong effect of CuL_2_phen in entry was further exploited on the attachment stage of SARS-CoV-2 entry in which the virus (MOI=1), and the compound were initially co-incubated with host cells at 4 °C for 1 h (Fig. 3g). At this lower temperature, the virus particles can attach to cellular receptors, but no membrane fusion occurs [80,82]. The data from this experiment revealed significant inhibitory effects of CuL_2_phen, showing an inhibition of 63.2% (Fig. 3h, *P*<0.0001). Interestingly, when an additional 30-min incubation at 37 °C was included in the previous protocol, a similar inhibition of 64.3% for CuL_2_phen was observed (Fig. 3i, j, *P* < 0.0001).

**Fig. 4.**
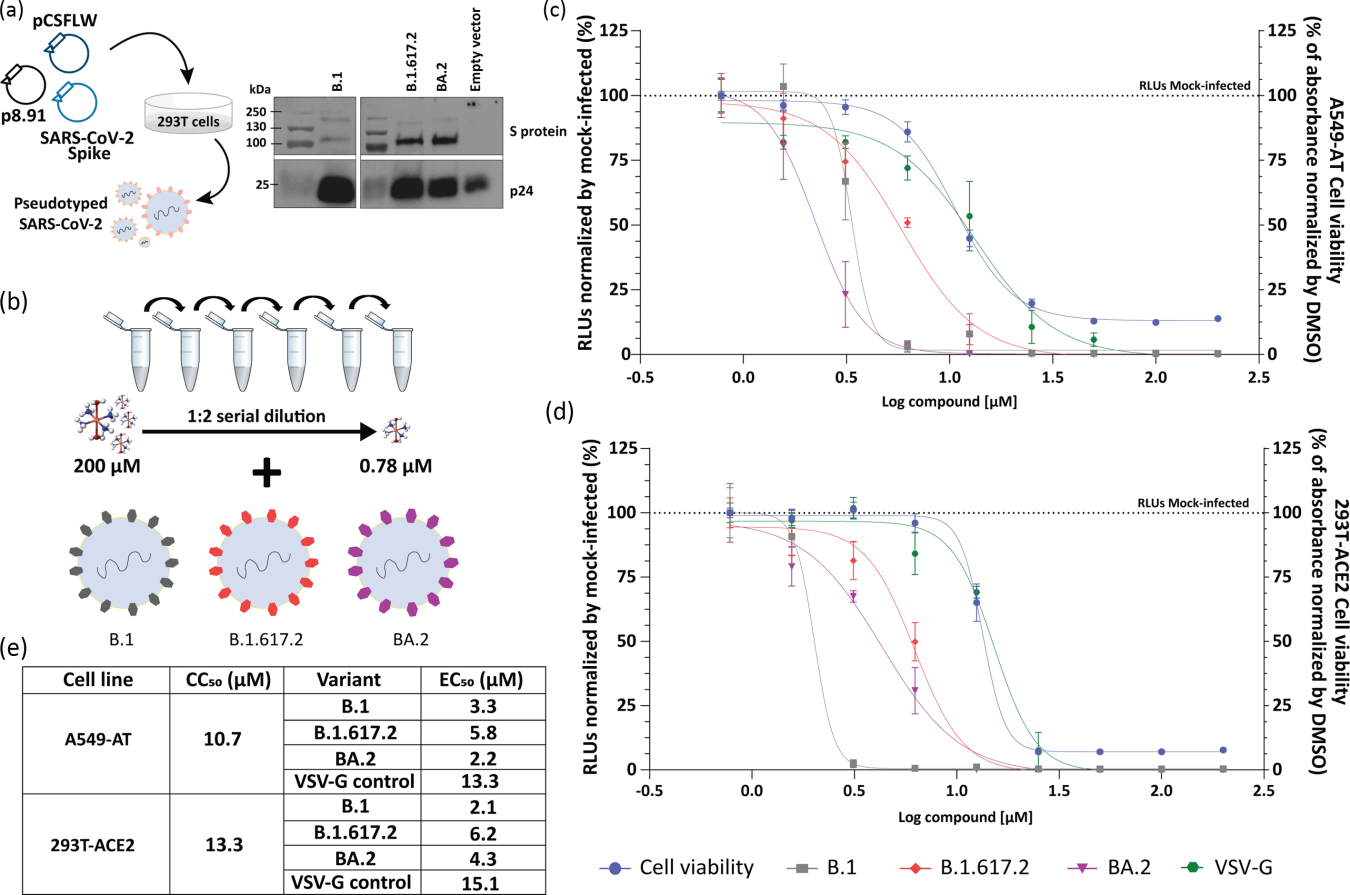
CuL2phen impairs SARS-CoV-2 pseudotyped virus entry. (**a**) Schematic representation of a lentiviral-based pseudotype for SARS-CoV-2. The plasmids p8.91 HIV Gag-Pol, pCSFLW luciferase and pcDNA3.1 carrying the S protein from SARS-CoV-2 B.1, B.1.617.2 or BA.2 were transfected into HEK293T cells. The empty vector (pcDNA3.1) and VSV-G were used as negative and positive pseudotyping control, respectively. The viral supernatant was collected at 48 h and 72 h post-transfection, and an aliquot was purified by ultracentrifugation in a 20% sucrose cushion and probed in a Western blot with anti-HIV p24 protein antibody (24 kDa) as lentivirus internal control as well as anti-S protein 1 A9 antibody (S and S1/S2 cleaved variant, 200 and 100 kDa, respectively). pcDNA3.1 was used as an empty vector. (**b–d**) Entry assay was performed by treating B.1, B.1.617.2 or BA.2 pseudotyped virus with a CuL_2_phen serial dilution (0.78 to 200 µM) for 1 h and subsequently adding to A549-AT (**c**) and 293T-ACE2 cells (**d**). The VSV-G was used as infection control. Viral entry was measured 72 h after infection by luciferase assay, while cytotoxicity was assessed by MTT assay as described previously. (**e**) The EC_50_, CC_50_ and SI for each cell line and variant are shown. The B.1, B.1.617.2 or BA.2 pseudotyped viruses are shown by the grey, red and purple colours in the schematic and graphs. Mean values±sd of a minimum of two independent experiments, each measured in quadruplicate. All images were generated using GraphPad Prism 9 and Adobe Illustrator 2025.

To further investigate the CuL_2_phen effect on SARS-CoV-2 entry, we produced pseudotyped SARS-CoV-2 carrying the B.1 spike protein or from two divergent variants B.1.617.2 and BA.2 (Fig. 4a) and employed those to perform an entry assay with CuL_2_phen and A549-AT or 293T-ACE2 cells (Fig. 4b). Interestingly, even after 72-h treatment, CuL_2_phen had a CC_50_ of 10.7 and 13.3 µM for A549-AT and 293T-ACE2, respectively. The inhibition curves and the EC_50_ for each variant in both cell lines are shown in Fig. 4c–e, which demonstrates that the entry of ancestral SARS-CoV-2 B.1 was affected by CuL_2_phen treatment with a similar EC_50_ to the SARS-CoV-2-NeonGreen. For the variants B.1.617.2 and BA.2, the compound was able to impair their entry into cells; however, both had higher EC_50_, suggesting a resistance to treatment. The compound was not able to inhibit VSV-G control entry, highlighting the effect of CuL_2_phen on inhibiting SARS-CoV-2 entry by interaction with S protein.

### Post-entry stages of SARS-CoV-2 infection are strongly inhibited by CuL_2_phen

**Fig. 5.**
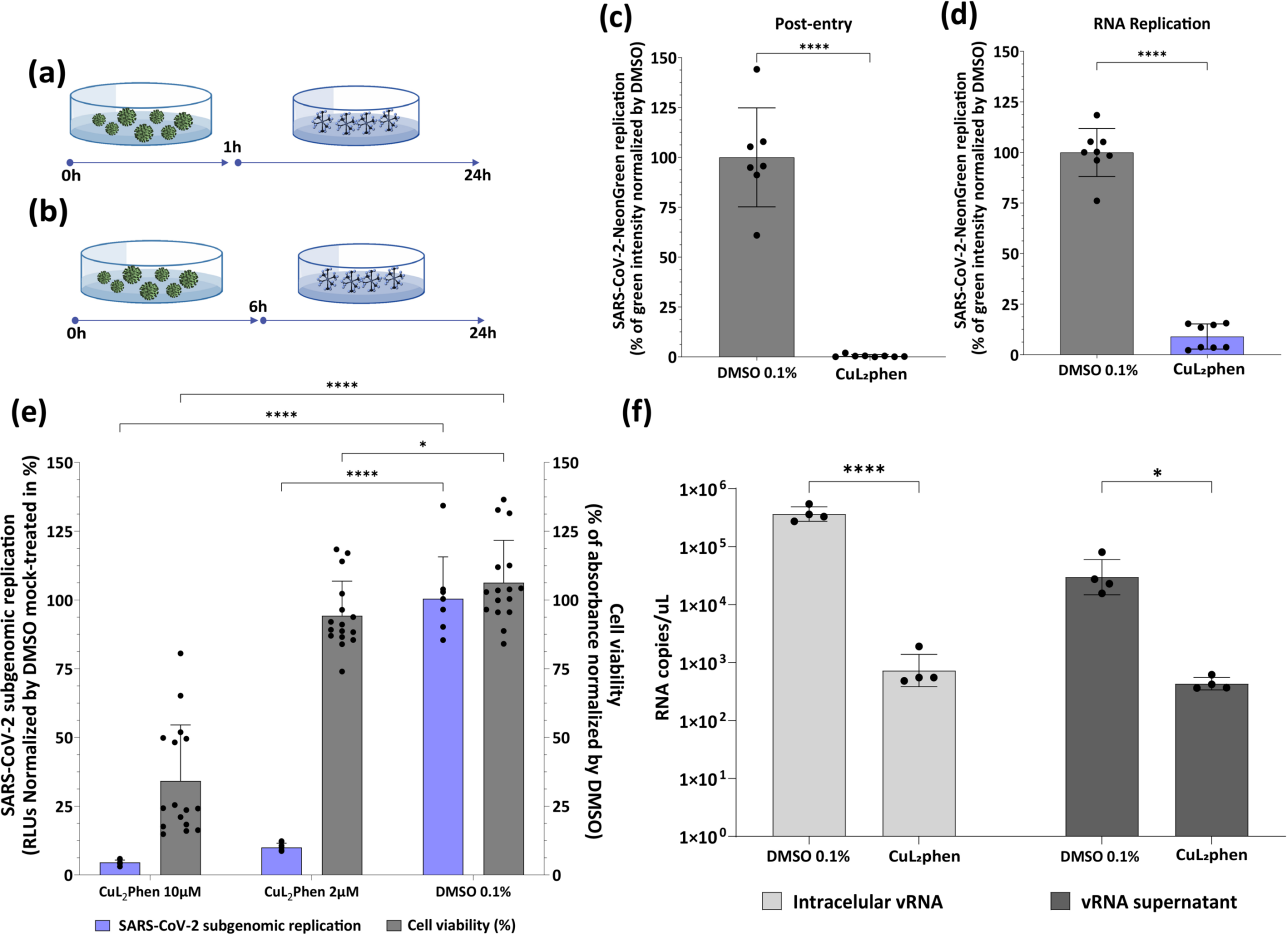
CuL_2_phen impaired post-entry stages of SARS-CoV-2 replication. A549-AT cells were infected with SARS-CoV-2-NeonGreen (MOI 1) for 1 h (**a**) or for 6 h (**b**) and then treated with CuL_2_phen at 2 µM for the remaining 24 h. Cells were washed and replaced with fresh media. For all assays, the total integrated intensity of the fluorescence (GCU×µm^2^ per well) was analysed using the IncuCyte S3 microscope, and post-entry and RNA replication are shown (c and d, respectively). The effect of CuL_2_phen on subgenomic RNA replication and cell viability was measured by transfecting BHK-21 cells with SARS-CoV-2-Repl-PL-NLuc, followed by treating the cells with the compound at 10 and 2 µM for 72 h (**e**). Replication was measured by luminescence levels and cell viability by MTT assay. Values obtained for treated samples were normalized to those of DMSO 0.1% treated samples. Quantification of viral RNA in supernatant and cell lysates from mock and CuL_2_phen-treated samples was performed by qPCR. Values were analysed by comparison to a standard SARS-CoV-2 RNA curve, and results are shown as RNA copies per microlitre (**f**). Schematic representation of each time-based assay as indicated by A549-AT cells (blue arrows), compounds (chemical structure) and SARS-CoV-2-NeonGreen (green virus). Mean values±sd of a minimum of three independent experiments, each measured in triplicate. Statistical differences were calculated by Student's t-test comparing DMSO mock-treated vs*.* CuL_2_phen. ns, non-significant, (*) *P*<0.05, (****) *P*<0.0001. All images were generated using GraphPad Prism 9 and Adobe Illustrator 2025.

**Fig. 6.**
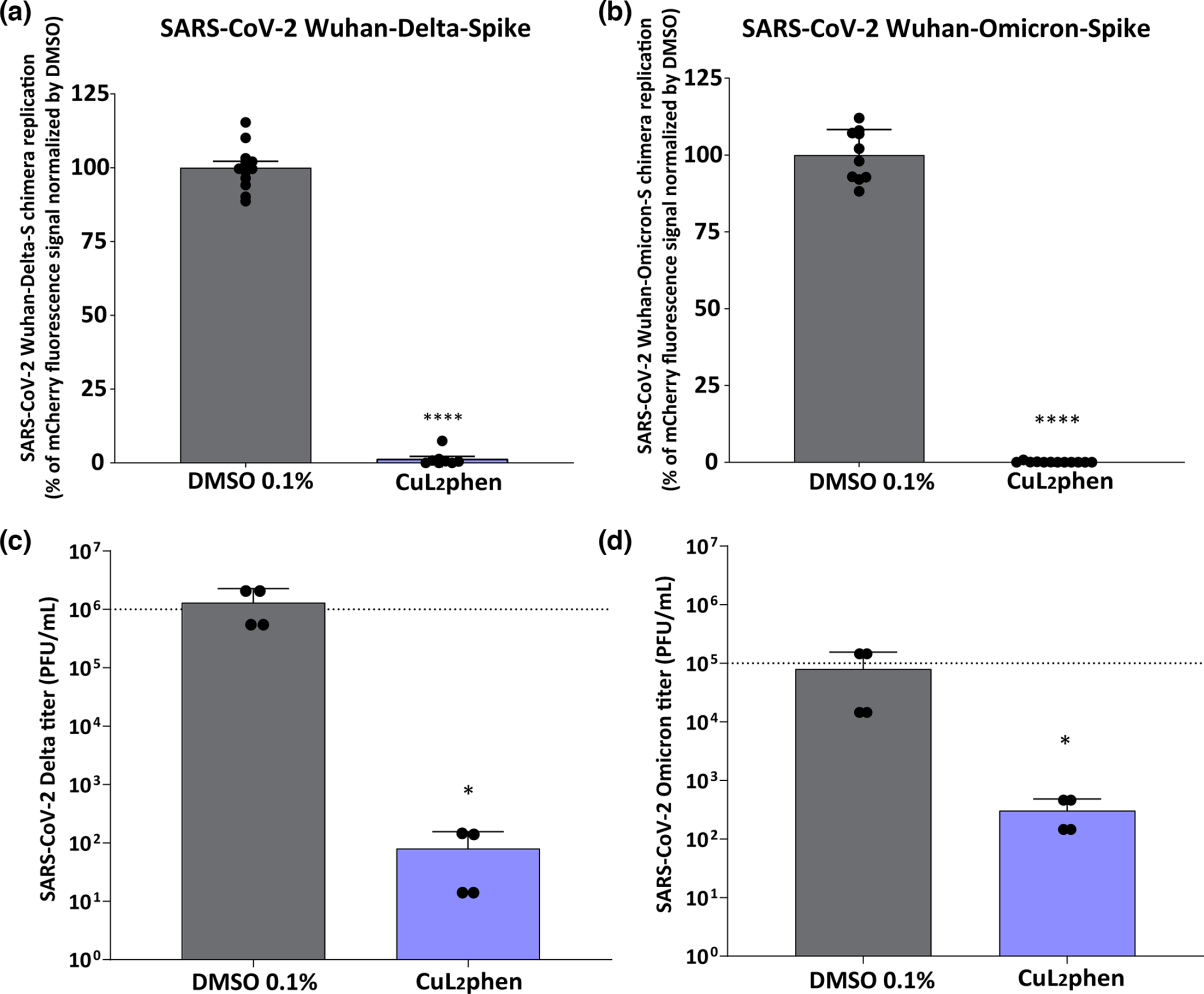
Effect of CuL_2_phen in SARS-CoV-2 variants. A549-AT cells were infected with SARS-CoV-2-Wuhan-B.1.617.2-Spike (**a**) or SARS-CoV-2-Wuhan-BA.2-Spike (**b**) at an MOI of 0.1 in the presence of CuL_2_phen at 2 µM for 24 h. SARS-CoV-2 replication was measured by the total integrated intensity of the fluorescence (RCU×µm^2^ per well) using the IncuCyte S3 microscope. To evaluate the wild-type variants, A549-AT cells were infected with SARS-CoV-2-B.1.617.2 (**c**) or SARS-CoV-2-BA.2 (**d**) at an MOI of 0.1 in the presence of CuL_2_phen at 2 µM for 24 h. Then, the supernatant was collected and titred by TCID_50_. All images were generated using GraphPad Prism 8 and Adobe Illustrator 2025. Mean values±sd of a minimum of three independent experiments, each measured in triplicate. Statistical differences were calculated by Student's t-test comparing DMSO mock-treated vs. CuL2phen. ns, non-significant, (*) *P*<0.05, (****) *P*<0.0001.

To further investigate the effect of CuL_2_phen on the SARS-CoV-2 replicative cycle, a post-entry assay was carried out to assess its impact on RNA replication and translation. Cells were infected with SARS-CoV-2-NeonGreen (MOI of 0.1) for 1 h (Fig. 5a) or 6 h (Fig. 5b), followed by the addition of media containing the compound. As a result, CuL_2_phen inhibited 99.6% of SARS-CoV-2-NeonGreen when treated immediately after 1 h of infection (Fig. 5c, *P*<0.0001) and maintained its inhibitory effect when added 6 h after viral infection, decreasing the RNA replication by 91% (Fig. 5d, *P*<0.0001).

Due to the strong inhibition on post-entry stages, the effect of CuL_2_phen was further assessed by transfecting a SARS-CoV-2 subgenomic RNA replicon in BHK-21 cells and then treating with CuL_2_phen at 10 µM and 2 µM, while simultaneously performing a cell viability assay. As a result, the compound at 2 µM had no statistical effect on cell viability compared to DMSO 0.1% (*P*>0.05) and reduced 90.1% of RNA replication and translation (Fig. 5e, *P*<0.0001). This was further confirmed by the quantification of viral RNA in both cell lysate and supernatant from A549-AT cells infected with SARS-CoV-2 (Wuhan-like – hCov-119/England/02/2020) by qPCR after treatment with CuL_2_phen. SARS-CoV-2 RNA levels in cell lysate and supernatant were reduced by 2.66 log₁₀ and 1.60 log₁₀, respectively, compared to mock-treated (Fig. 5).

### SARS-CoV-2 B.1.617.2 and BA.2 variants are strongly inhibited by CuL_2_phen

The evolution of SARS-CoV-2 and the emergence of highly divergent variants necessitate the development of antiviral compounds that can broadly inhibit viral replication independently of the accumulation of mutations [4,83,85]. In this context, the CuL_2_phen effect was confirmed against the Delta and Omicron variants, by first employing infectious clones of the Wuhan strain with the Spike coding sequences from the B.1.617.2 (Delta) or BA.2 (Omicron) variants carrying the mCherry gene engineered by Rihn and coworkers [57] following a similar protocol to Fig. 3 and quantified by mCherry fluorescence (RCU×µm^2^ per well) as previously reported [49]. The results showed that CuL_2_phen led to a reduction of 98.7% in SARS-CoV-2 Wuhan-B.1.617.2-Spike (Fig. 6a, *P*<0.0001), while when challenged against the SARS-CoV-2 Wuhan-BA.2-Spike virus, CuL_2_phen was able to suppress viral replication by 99.9% (Fig. 6b, *P*<0.0001). This suggests that the mutations accumulated within the S glycoprotein were not able to induce resistance to CuL_2_phen and emphasizes that its inhibition would be related to replication steps rather than viral entry.

To further validate these data, wild-type variants B.1.617.2 (Delta) and BA.2 (Omicron) were employed to examine their impact on viral replication following the same protocol but assessing the viral titres in supernatant using the TCID_50_ method. The results demonstrated that against the Delta variant, CuL_2_phen led to a decrease in viral titre of 4 Log _10_(Fig. 6c). Similarly, for the Omicron variant, CuL_2_phen inhibited viral replication by ~2.5 Log_10_ (Fig. 6d).

### Insights into the mode of action

#### CuL_2_phen interacts with SARS-CoV-2 particles

**Fig. 7.**
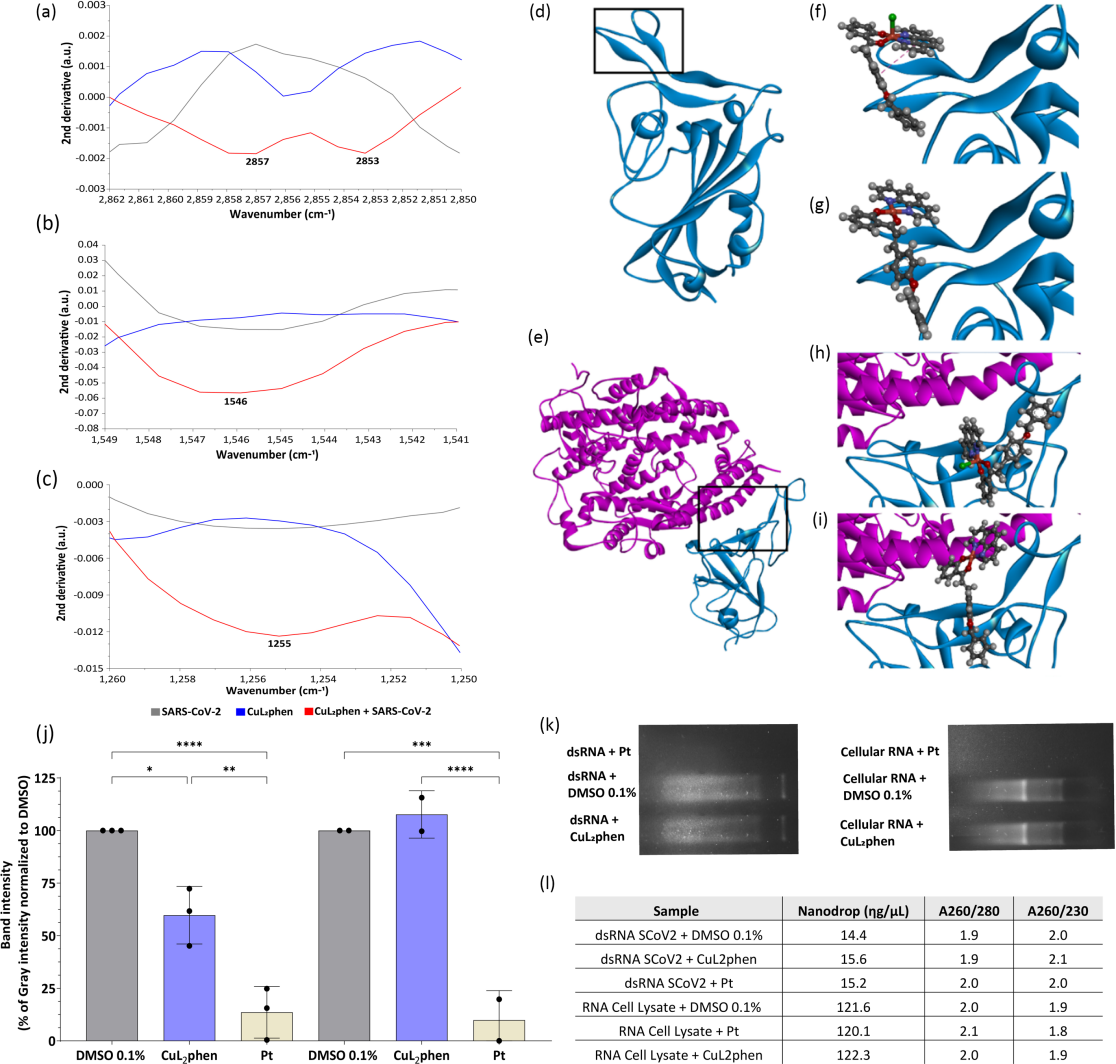
Insights into the mechanisms of action of CuL_2_phen on SARS-CoV-2. Representative scheme of the ATR-FTIR technology with SARS-CoV-2 (grey line), CuL_2_phen (blue line) and SARS-CoV-2 incubated with CuL_2_phen (red line). The spectra are depicted in panels (**a**–**c**). SARS-CoV-2-RBD extracted from PDB 7E86 (**d**) and SARS-CoV-2-RBD-ACE2 (**e**) and prepared by Discovery Studio Visualizer v21.1.0.20298 software prior to docking. The main area of the best poses of docking is highlighted (black square). SARS-CoV-2-RBD docked [CuL_2_phen] (**f**) and [CuL_2_phen]^+^ (**g**). SARS-CoV-2-RBD-ACE2 docked [CuL_2_phen] (**h**) e [CuL_2_phen]^+^ (**i**). Sixty-five nanomoles of SARS-CoV-2 ORF1a dsRNA were incubated with CuL_2_phen at 2 µM or *cis*-[PtCl_2_(dmso)_2_] (Pt) at 10 µM or the DMSO 0.1% control for 45 min at room temperature. RNA extracted from untreated A549-AT cells was also incubated with CuL_2_phen, Pt or DMSO 0.1%. The reaction products were subjected to 1% agarose 1X TAE electrophoresis gel containing SYBR safe, followed by densitometry analysis on ImageJ.JS version 1.53 j, which is shown in (**j**) and (**k**), respectively. Bands were extracted, purified and quantified to check the RNA integrity, which is shown in panel (**l**). Mean values±sd of a minimum of two independent experiments. Statistical differences were calculated by Student's t-test by comparing DMSO mock-treated vs. CuL_2_phen or Pt. (*) *P*<0.05, (**) *P*<0.01, (****) *P*<0.0001. The figure was generated with Adobe Illustrator 2025.

The ATR–FTIR spectroscopy allows the identification of a bio-fingerprint in the spectral region between 800 and 1,900 cm^−1^ [86,88]. In this region, it is possible to characterize a virus-specific vibrational mode, and any change in the spectral profile indicates alteration in molecular vibrations associated with lipids, carbohydrates, proteins, amide II or amide III [89,92]. The comparison of the infrared spectra of SARS-CoV-2, CuL_2_phen and SARS-CoV-2 incubated with CuL_2_phen for 1 h demonstrated molecular changes in SARS-CoV-2 spectra at the bio-fingerprint region (1,800–900 cm^−1^), suggesting potential interactions primarily involving four vibrational modes: at 2,857 cm^−1^ and 2,853 cm^−1^ (Fig. 7a), assigned to lipid (symmetric and asymmetric stretch of acyl CH2 groups); 1,546 cm^−1^ (Fig. 7b) related to the amide II band (N–H stretch) and 1,255 cm^−1^ (Fig. 7c) correspondent to amide III vibrational mode [62,93,95]. Interestingly, the change in these vibrational modes was detected only after incubation of SARS-CoV-2 particles with the CuL_2_phen and was not detected in SARS-CoV-2 or CuL_2_phen alone, suggesting significant interactions with the viral surface.

Based on the FTIR data, the interaction of CuL_2_phen with the SARS-CoV-2 RBD was investigated since it has a critical role in binding to host cells via the ACE2 receptor [96,97]. To gain insights into these interactions, molecular docking predictions were performed using the RBD (Fig. 7d) and the co-crystallized RBD–ACE2 complex (Fig. 7e). The crystal structure determination of CuL_2_phen, previously reported, shows an apical chloride coordinated moiety [48], and since the chloride was demonstrated to dissociate in solution, prompting us to perform the docking analysis for both CuL_2_phen and [CuL_2_phen]^+^.

Although the loss of chloride results in a positive charge on the complex, it is important to highlight that GOLD, the docking tool used, primarily considers steric effects and Van der Waals interactions, ignoring atomic and molecular charges. This limitation prevents the evaluation of charge effects in the analysis. To assess this limitation’s impact, we checked the charge distribution for both RBD and RBD–ACE2, revealing mostly neutral surfaces (Fig. S1).

The highest-scoring poses suggest potential binding modes for the complexes with significant interactions. In the RBD structure, both CuL_2_phen (Fig. 7f) and [CuL_2_phen]^+^ (Fig. 7g) show favourable interactions with notably high scores (Table 1). CuL_2_phen engages in *π*-*π* stacking with the PHE456 residue through the *α*-ring of the L2, while [CuL_2_phen]^+^ interacts through L2 with the same PHE456 residue, but via the chalcone *β*-ring. This allows the *α*-ring to interact with LEU455 through a *π*-alkyl interaction and LYS417 (Fig. S2). Additionally, in the latter pose, the phenanthroline (phen) ligand interacts via *π*-stacking with TYR489 and GLY485, resulting in a higher score. A substantial difference between the poses is observed, with CuL_2_phen bending over, suggesting intramolecular *π*-*π* stacking (Fig. 7h, i). This result indicates that chloride may impose steric restrictions, reducing the number of contacts. Given the experimental evidence of chloride loss in solution, we suggest that the interaction of [CuL_2_phen]^+^ is more relevant.

**Table 1.** CHEMPLP fitness score and interacting residues for the best poses obtained in the molecular docking for [CuL2(phen] and [Cu(BC)(phen)]^+^ in SARS-CoV-2-RBD and SARS-CoV-2-RBD-ACE2

RBD		
Compound	CHEMPLP score	Interacting residues
[Cu(L_2_)(phen)Cl]	63.5233	PHE456
[Cu(L_2_)(phen)]^+^	67.3255	LYS417, LEU455, PHE456, GLY485, TYR489
**RBD–ACE2**		
**Compound**	**CHEMPLP** **score**	**Interacting residues**
[Cu(L_2_)(phen)Cl]	68.0823	GLY482, CYS480, CYS488, ILE472, GLY484, PHE490, THR470, LEU492
[Cu(L_2_)(phen)]^+^	75.8641	PHE28, LYS31, TYR489

RBD, Receptor binding domain.

The resulting poses for the compounds with the RBD–ACE2 complex also indicate favourable interactions with high scores. CuL_2_phen engages in *π*-type interactions solely with residues of the RBD subunit, while [CuL_2_phen]^+^ interacts with three ACE2 receptor residues and two RBD residues, probably due to the restrictions imposed by chloride steric hindrance.

Energy calculations using the Molecular Mechanics/Generalized Born Surface Area (MM/GBSA) approximation reveal that the binding free energy of the RBD subunit with the ACE2 receptor, in the absence of metal complexes, is −29.18 kcal mol^−1^, while with CuL_2_phen or [CuL_2_phen)]^+^ docked, this energy increases to −27.39 kcal mol^−1^ and −28.87 kcal mol^−1^, respectively, indicating that the presence of the complexes reduces the interaction between the RBD subunit and the ACE2 receptor, suggesting one possible mode of action.

### CuL_2_phen interacts with SARS-CoV-2 dsRNA

Considering the potent post-entry effect on SARS-CoV-2 replication, the mode of action of CuL_2_phen was further investigated by a dsRNA interaction assay. Given the essential role of dsRNA in positive-sense RNA virus replication, this could be considered the potential target [98]. Utilizing the ORF1a region of SARS-CoV-2 as a template, a dsRNA molecule of 499 bp was synthesized and incubated with CuL_2_phen or with the controls (DMSO 0.1% and *cis*-[P*t*C*l*_2_(*dmso*)_2_] at 10 µM). Further, cellular RNA was also incubated with CuL_2_phen or with the controls to confirm the compound’s specificity to a dsRNA target. Through a migration retardation assay, CuL_2_phen was observed to reduce 41% of the band intensity in the gel compared to the negative control DMSO (*P*<0.05), as quantified by densitometry (Fig. 7j, k), suggesting an interaction between CuL_2_phen and dsRNA. Interestingly, the compound did not show any statistical effect on RNA extracted from cells, demonstrating a specific interaction with viral dsRNA (Fig. 7j). It is noteworthy that no band can be observed in the gel for the samples treated with *cis*-[PtCl_2_(dmso)_2_] (*P*<0.01), which serves as a positive control for dsRNA interaction (Fig. 7j). However, to confirm that this interaction did not result from RNA degradation, the bands were extracted and quantified by spectrometry, which demonstrated no degradation or changes in RNA concentration after incubation with the compound (Fig. 7l). This result further supports the antiviral findings obtained.

## Discussion

The compounds CuL_2_phen and CuL_1_phen were synthesized and screened against SARS-CoV-2; however, the descriptive antiviral profile and potential of these new metal complex species were not further characterized [48]. Here, our data showed that CuL_2_phen presented a better SI than CuL_1_phen (values of 5.3 and 1.7, respectively). The fluorination (addition of a fluorine atom) in the chalcone *α*-ring in the compound CuL_1_phen is known to decrease the ligand substitution rate, therefore, resulting in a higher cytotoxicity by impairing the speciation of the molecule in biological systems [99,100]. Additionally, CuL_1_phen was shown to have a stronger cytotoxic effect when evaluated against *Leishmania* sp*.* and human cells, with a lower selective effect against the parasite when compared to CuL_2_phen [48]. This agrees with data presented here, since CuL_1_phen showed a lower CC_50_ when compared with the non-fluorinated compound CuL_2_phen.

Cu(II) plays crucial roles in various cellular processes in the human body, being essential for enzymatic activity, iron metabolism, DNA synthesis and repair and cell growth and development [101,105]. In this study, CuL_2_phen demonstrated remarkable anti-SARS-CoV-2 activity, with EC_50_ and CC_50_ that resulted in a more selective activity, particularly in impairing the post-entry steps of SARS-CoV-2. This suggests the potential for achieving superior antiviral outcomes even at lower concentrations.

Notably, CuL_2_phen exhibited robust effectiveness against the SARS-CoV-2 B.1.617.2 and BA.2 variants, reducing viral titres by over 2.5 Log_10_ after treatment [106,108]. Another point to be considered is that the modest SI of CuL_2_phen *in vitro* might be overruled by the lack of adverse effects *in vivo* when considering therapeutic significance. However, it is imperative to underscore that the effective concentration for the treatment of infected animals and/or individuals requires further evaluation. Other drugs, such as chloroquine, were described with high SI *in vitro* and did not show any therapeutic significance when evaluated *in vivo* and in clinical trials [106,109].

Cu(II) ions were described as able to generate reactive oxygen species (ROS) in mammalian cells [41,45]. Previously, the Cu(II) complexes described here were also characterized as capable of speciation in biological systems, probably releasing the Cu(II) ions [48]. In addition, A549 cells are capable of producing ROS and inducing IL-8 mRNA, which are essential to inhibit SARS-CoV-2 dissemination in human infection [110,111]. Our data demonstrated a modest but significant protection of cells against SARS-CoV-2 infection, which might be explained by the activation of ROS pathways, decreasing the infection by degrading part of the viral particles as soon as they enter the cells. However, due to the strong post-entry inhibition, it is possible that the modest protection observed is rather a secondary effect than a true cell protection. Furthermore, CuL_2_phen demonstrated to be a potent entry inhibitor, which might also be related to the chemical speciation, releasing Cu(II) molecules that can disrupt the viral envelope [37,41, 42]. This was emphasized by the effect on SARS-CoV-2 pseudotyped viruses in association with the FTIR data, demonstrating the direct interaction of the compound with the viral particle. The stretch in the vibrational modes at 2,857 cm^−1^, 2,853 cm^−1^, 1,546 cm^−1^ and 1,255 cm^−1^ represents the lipid and amide groups present in the SARS-CoV-2 membrane particles, in which 1,546 cm^−1^ and 1,255 cm^−1^ were associated with N and S proteins, respectively [112]. With these results, it is possible to suggest that CuL_2_phen is also impairing SARS-CoV-2 entry by directly interacting and/or disrupting viral structure. This was further suggested by the molecular docking, which showed significant interaction of the CuL_2_phen with the SARS-CoV-2-RBD, decreasing the interaction of the RBD-ACE2 by 1.2 kcal mol^−1^. As these proteins present a hydrophobic surface, the *π*-stacking interaction of the ligands and the protein is favoured.

CuL_2_phen was identified as a stronger inhibitor of viral replication, which agrees with previous reported effects of both chalcones and Cu(II) in combination with host factors and viral proteins resulting in impaired viral replication [43,45, 113, 114]. Since Cu(II) complexes were described to interact with nucleic acids [43,115, 116], we hypothesized that one of its targets could be dsRNA, which was confirmed by a direct intercalation assay in which CuL_2_phen-SARS-CoV-2-dsRNA incubation resulted in a lower intensity band on an agarose gel. This interaction directly impacts viral RNA replication due to the necessity of the complementary negative-sense RNA to produce subgenomic mRNA that encodes structural and accessory proteins, besides the production of new viral RNAs [3,98]. In addition, it is noteworthy that chalcone molecules have previously demonstrated their potential as inhibitors of SARS-CoV 3CL^pro^ cysteine proteinase and the replication of a subgenomic replicon for hepatitis C virus RNA [18,117,120]. Herein, CuL_2_phen was able to decrease subgenomic replication in cells transfected with SARS-CoV-2 subgenomic replicon, opening up the intriguing possibility that CuL_2_phen could be influencing the viral replication proteins and that Cu(II) could be serving as a carrier for L_2_, effectively functioning as a delivery method. This theory gains support from the observation that L_2_ alone did not inhibit SARS-CoV-2 [48].

CuL_2_phen has demonstrated interesting efficacy in suppressing SARS-CoV-2 infection *in vitro* by interacting with the SARS-CoV-2 virion, especially focused on the S glycoprotein but also interacting with SARS-CoV-2 dsRNA and impairing post-entry viral replication. The effect of CuL_2_phen was also confirmed by the inhibition of B.1.617.2 and BA.2 variants. Based on these promising results, we advocate for the initiation of further investigations involving Cu(II) complexes. Furthermore, we propose that CuL_2_phen be considered for utilization in pre-clinical assays as a promising alternative for the treatment of COVID-19.

## Supplementary material

10.1099/jgv.0.002245 Supplementary Material 1.

## Abbreviations

ACE2: angiotensin-converting enzyme 2
ATR: attenuated total reflection
CC_50_: cytotoxic concentration of 50%
COVID-19: coronavirus disease 2019
DMEM: Dulbecco’s modified Eagle’s Medium
d.p.i.: days post-infection
EC_50_: effective concentration of 50%
FTIR: Fourier transform infrared
GA: genetic algorithm
GA: genetic algorithm
MM/GBSA: Molecular Mechanics/Generalized Born Surface Area
MOI: multiplicity of infection
MTT: 3-(4,5-dimethylthiazol-2-yl)-2,5-diphenyl tetrazolium bromide
NCBI: National Center for Biotechnology Information
NIBSC: National Institute for Biological Standards and Control
ORF: Open reading frame
PVs: pseudotyped viruses
qPCR: quantitative PCR
RBD: receptor-binding domain
RLU: relative luminescence units
ROS: reactive oxygen species
SARS-CoV-2: severe acute respiratory syndrome coronavirus 2
SI: selective index
