# Supplementary material for: Copper(II) benzyloxychalcone analogues as new potential metallodrugs against SARS-CoV-2 replication
Source: J Gen Virol. 2026 May 5;107(4):002245. doi: 10.1099/jgv.0.002245 (PMC13141362; doi:10.1099/jgv.0.002245)
Supplement: Supplementary Material 1. [file jgv-107-02245-s001.pdf]

Supplementary Figures

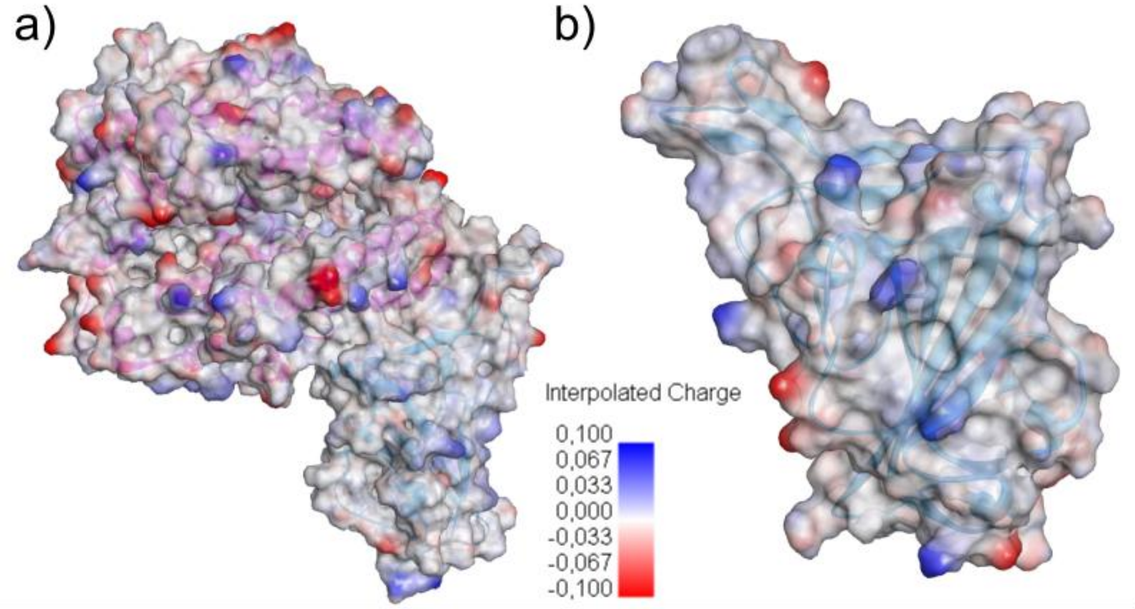

Figure S1. Surface charge distribution for SARS-CoV-2-RBD-ACE2 (a) and SARS-CoV-2-RBD (b)

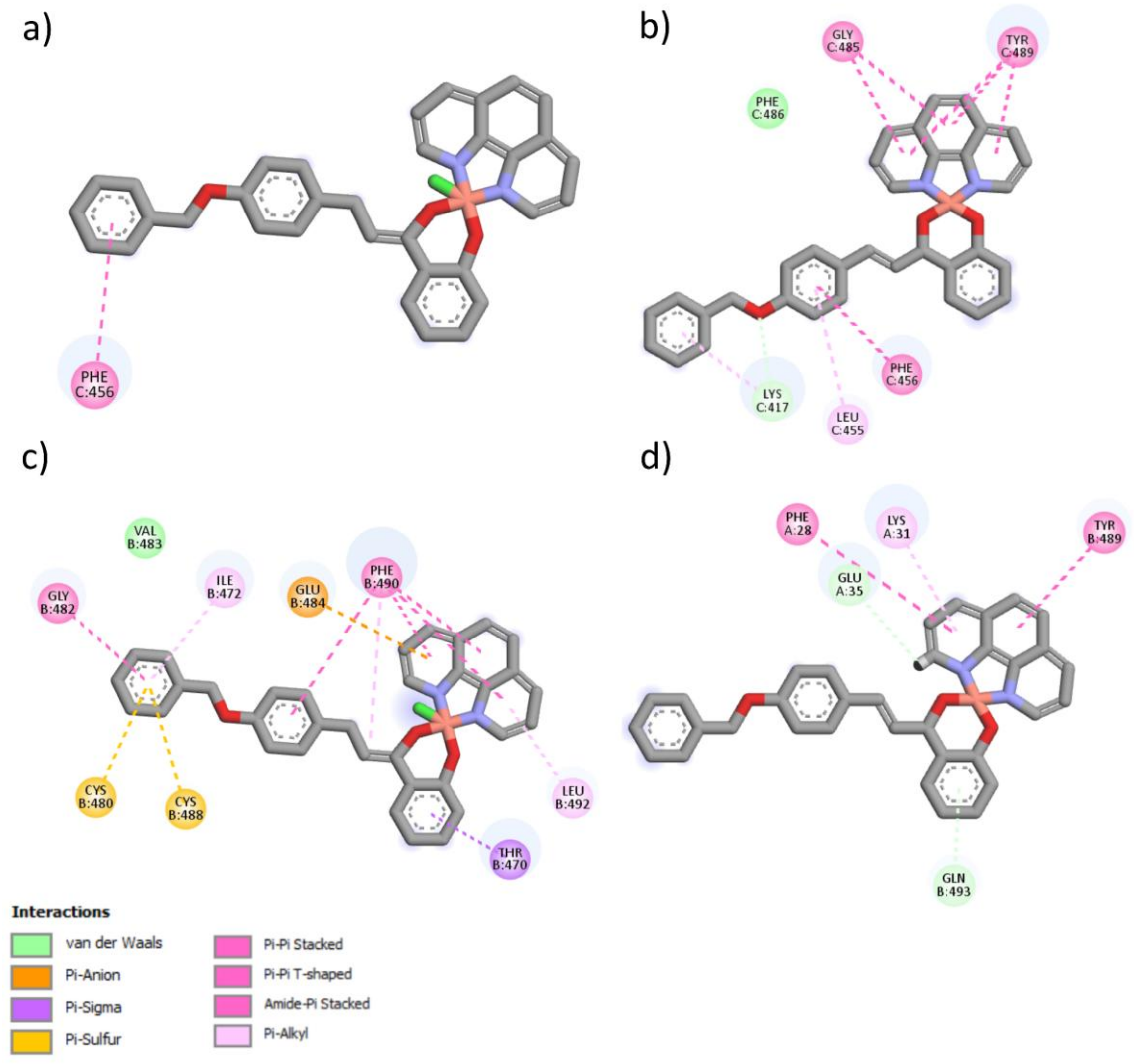

Figure S2. Residues interactions for the best docking poses for isolated RBD with CuL$_2$phen (a) and [CuL2phen]$^+$(b) and RBD-ACE2 with CuL$_2$phen (c) and [CuL$_2$phen]$^+$ (d).

Supplementary Table 1: Mutations encountered in our SARS-COV-2 Wuhan-like stock after sequencing. Synonymous mutations are in italic.

**Genome Position**	**Nucleotide at Genome Reference**	**Mutated nucleotide**	**Mutation Frequency**	**SARS-CoV-2 region**	**Condon at Genome Reference**	**Amino acid at Genome Reference**	**Alternative Condon**	**Alternative Amino acid (mutated)**	**Amino acid postion at the protein**
435	A	G	4%	ORF1ab	GAA	E	GGA	G	57
2198	G	A	16%	ORF1ab	GGT	G	AGT	S	645
2236	T	A	13%	ORF1ab	TGT	C	TGA	*(stop codon)	657
3540	C	T	13%	ORF1ab	GCT	A	GTT	V	1092
3903	C	T	21%	ORF1ab	CCA	P	CTA	L	1213
5736	C	T	29%	ORF1ab	GCT	A	GTT	V	1824
6078	C	T	19%	ORF1ab	GCT	A	GTT	V	1938
6255	C	T	12%	ORF1ab	GCA	A	GTA	V	1997
6884	G	A	11%	ORF1ab	GGT	G	AGT	S	2207
7472	G	A	17%	ORF1ab	GGT	G	AGT	S	2403
8380	A	G	3%	ORF1ab	AAA	K	AAG	K	2705
8782	*C*	*T*	*97%*	*ORF1ab*	*AGC*	*S*	*AGT*	*S*	*2839*
9246	C	T	11%	ORF1ab	GCT	A	GTT	V	2994
10440	C	T	10%	ORF1ab	GCT	A	GTT	V	3392
10834	*C*	*T*	*11%*	*ORF1ab*	*GCC*	*A*	*GCT*	*A*	*3523*
11669	C	T	15%	ORF1ab	CGC	R	TGC	C	3802
11991	A	G	4%	ORF1ab	GAA	E	GGA	G	3909
12119	C	T	36%	ORF1ab	CCA	P	TCA	S	3952
13476	*C*	*T*	*21%*	*ORF1ab*	*TGC*	*C*	*TGT*	*C*	*3*
13762	G	A	13%	ORF1ab	GGT	G	AGT	S	99
16838	A	G	4%	ORF1ab	GAA	E	GGA	G	1124
16987	G	A	12%	ORF1ab	GGC	G	AGC	S	1174

17196	G	A	13%	ORF1ab	AAG	K	AAA	K	1243
17440	C	T	7%	ORF1ab	CCT	P	TCT	S	1325
17567	G	A	13%	ORF1ab	TGT	C	TAT	Y	1367
17845	C	T	11%	ORF1ab	CAG	Q	TAG	*(stop codon)	1460
18488	T	C	98%	ORF1ab	ATA	I	ACA	T	1674
19484	C	T	16%	ORF1ab	GCT	A	GTT	V	2006
21005	C	T	19%	ORF1ab	GCA	A	GTA	V	2513
22296	A	G	16%	S	CAT	H	CGT	R	245
23605	*T*	*G*	*83%*	*S*	*CCT*	*P*	*CCG*	*P*	*681*
23616	G	A	88%	S	CGT	R	CAT	H	685
23874	C	T	25%	S	GCT	A	GTT	V	771
24245	C	T	11%	S	CAA	Q	TAA	*(stop codon)	895
25770	G	A	15%	ORF3a	AGG	R	AGA	R	126
25964	A	G	3%	ORF3a	GAA	E	GGA	G	191
26261	C	T	4%	E	TCG	S	TTG	L	6
26354	T	A	38%	E	CTT	L	CAT	H	37
26753	C	T	14%	M	ACC	T	ACT	T	77
28144	T	C	95%	ORF8	TTA	L	TCA	S	84
28531	*C*	*T*	*6%*	*N*	*TAC*	*Y*	*TAT*	*Y*	*86*
28916	G	A	15%	N	GGT	G	AGT	S	215
29366	C	T	19%	N	CCA	P	TCA	S	365
29596	A	G	96%	ORF10	ATA	I	ATG	M	13
